# Wet spinning imogolite nanotube fibres: an *in situ* process study†

**DOI:** 10.1039/d3na00013c

**Published:** 2023-05-31

**Authors:** Joseph F. Moore, Erwan Paineau, Pascale Launois, Milo S. P. Shaffer

**Affiliations:** a Department of Materials, Imperial College London Exhibition Road SW7 2AZ UK m.shaffer@imperial.ac.uk; b Université Paris-Saclay, CNRS, Laboratoire de Physique des Solides 91405 Orsay France; c Department of Chemistry, Imperial College London 82 Wood Lane W12 0BZ UK

## Abstract

Imogolite nanotubes (INTs) form transparent aqueous liquid-crystalline solutions, with strong birefringence and X-ray scattering power. They provide an ideal model system for studying the assembly of one-dimensional nanomaterials into fibres, as well as offering interesting properties in their own right. Here, *in situ* polarised optical microscopy is used to study the wet spinning of pure INTs into fibres, illustrating the influence of process variables during extrusion, coagulation, washing and drying on both structure and mechanical properties. Tapered spinnerets were shown to be significantly more effective than thin cylindrical channels for forming homogeneous fibres; a result related to simple capillary rheology by fitting a shear thinning flow model. The washing step has a strong influence of structure and properties, combining the removal of residual counter-ions and structural relaxation to produce a less aligned, denser and more networked structure; the timescales and scaling behavior of the processes are compared quantitatively. Both strength and stiffness are higher for INT fibres with a higher packing fraction and lower degree of alignment, indicating the importance of forming a rigid jammed network to transfer stress through these porous, rigid rod assemblies. The electrostatically-stabilised, rigid rod INT solutions were successfully cross-linked using multivalent anions, providing robust gels, potentially useful in other contexts.

## Abstract 2

*In situ* polarised optical microscopy, during coagulation spinning and washing, reveals the competing processes determining the mechanical properties of pure imogolite nanotube fibres.

## Graphical abstract

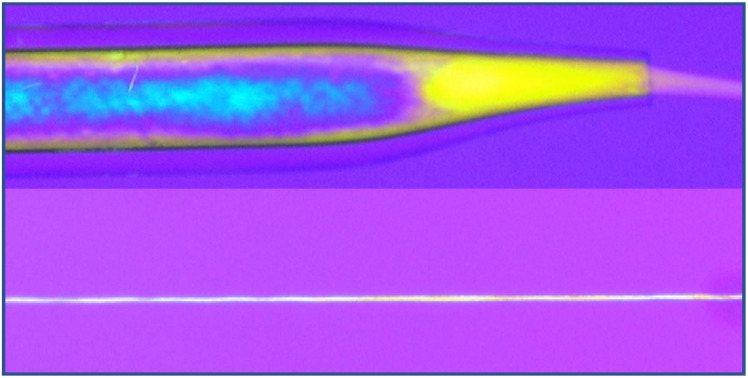

## Introduction

The excellent intrinsic mechanical properties of nanotubes, alongside their 1D morphology, motivates their use in structural and multifunctional fibres, either as pure assemblies or as composites.^1–4^ The mechanical properties of macroscale assemblies of nanotubes are typically limited by the weak interactions between the constituent nanotubes, which leads to poor stress-transfer and failure by inter-nanotube sliding, rather than breakage of individual nanostructures. Significant developments have been made in constructing fibres from carbon nanotubes (CNTs) by increasing the packing density and CNT aspect ratio to improve stress-transfer.^5–11^ However, analysing the structure, and in particular the degree of alignment, of CNT fibres is challenging due to their high optical absorbance and size-dispersity, alongside the low X-ray scattering cross-section of the carbon atoms. X-ray synchrotron diffraction is therefore often preferred for studying individual single-walled CNT fibres.^12^

Imogolite nanotubes (INTs) offer an opportunity to study the assembly of nanotubes into fibres using both polarised optical microscopy (POM) and lab-source X-ray scattering (XRS). Classic INTs have similar dimensions to single or double wall CNTs, based on an intrinsically curved aluminosilicate layer structure with hydroxylated inner and outer surfaces. In contrast to CNTs, INTs are optically transparent and can be synthesised with uniform diameters, which leads to sharper and more intense XRS features. The use of aluminogermanate variants increases the scattering strength further, due to the substitution of Si atoms with highly-scattering Ge atoms. INTs can be easily synthesised at relatively low temperature (100–150 °C) and improvements in synthesis methods have enabled the growth of micron length INTs which may be spun into fibres using a wet spinning approach;^13^ however, the spinning process is not yet optimised or fully understood.

INTs are known to form liquid crystalline dispersions in water spontaneously at a critical volume fraction which can vary with the INT aspect ratio, the ionic strength of the aqueous dispersant and even the choice of precursor used in the synthesis.^14–18^ Liquid crystalline spinning dopes are of great interest for wet spinning fibres due to the inherent opportunity to form highly aligned fibres, by reorienting the liquid crystalline domains during spinning. However, this reorientation is not guaranteed due to the complex behaviour of rigid rod suspensions in shear, with multiple possible motions ranging from log rolling, tumbling, wagging and finally shear aligning with increasing strain rates.^19^

The recent wet spinning process for pure INTs produced continuous fibres with promising properties.^13^ This work develops pure INT wet spinning, using *in situ* observation to understand the assembly mechanisms at each stage of the process. The aim was both to improve the properties of the INT fibres and to develop a mechanistic understanding potentially relevant to a variety of rigid rod nanomaterials.

## Experimental

### INT synthesis

Double-walled germanium INTs were synthesised by a hydrothermal method using aluminium perchlorate nonahydrate (Alfa Aesar), tetraethoxygermane (TEOG, Sigma-Aldrich) and urea (Sigma-Aldrich) following a procedure described elsewhere.^15^ Briefly, TEOG, aluminium perchlorate and urea were mixed in a PTFE beaker with a molar ratio of [Ge] : [Al] : [urea] = 1 : 2 : 2. The beaker was placed in a PTFE-lined acid digestion bomb and treated at 140 °C for 40 days to enable INT growth.^13^ The INT solutions were then dialysed against ultrapure water using a membrane (Spectra/Por, cutoff = 10 kDa) until the bath conductivity drops below 0.5 mS m^−1^. INT concentration was determined by heating 2 mL of the synthesised INT solution in an oven at 90 °C. The mass of the dry INT film was measured (22.1 ± 0.1 mg) to give a solution mass concentration of 11 mg mL^−1^. An aliquot (5 mL) of the stock solution was serially diluted to create solutions of 5.5, 3 and 1 mg_INT_ mL^−1^.

### Capillary rheometry

Simplified capillary rheometry was conducted under conditions related to the spinning process, by injecting INT solutions through PEEK capillary tubing (Trajan, inner diameter 178 μm, length 50 cm) with a syringe pump (KD Scientific, Legato 100) at flow rates between 0.025 and 2 mL h^−1^. The pressure drop over the capillary was measured by a piezoresistive pressure sensor (Honeywell, ABPDANT030PG0D3). The wall shear rate, *γ̇*_w_, was determined by plotting the log apparent (Newtonian) wall shear rate, *γ̇*_a,w_, *vs.* the log shear stress and finding the gradient, *b*, to apply the Rabinowitch correction, *γ̇*_w_ = (3 + *b*)/4*γ̇*_a,w_ as described elsewhere.^20^ The shear viscosity was determined as wall shear stress divided by wall shear rate.

### INT fibre spinning

In a typical experiment, INT fibres were prepared by injecting INT solution (11 mg_INT_ mL^−1^) at 5 mL h^−1^ through a tapered glass spinneret nozzle (taper semi-angle 8°, orifice diameter 451 μm) into a coagulation bath of aqueous CaCl_2_ (300 g_CaCl_2__ L^−1^) prepared from calcium chloride dihydrate (99%, MilliporeSigma). Tapered spinneret nozzles were prepared by necking soda glass capillary tubing (Samco G119/02) in a fine blow torch flame. The necked tubing was snapped off and the tips were gently ground flat using P1000 grit SiC abrasive paper and then flame polished. The proto-fibres were collected on a PTFE wheel (100 mm diameter) at a draw ratio (take-up velocity divided by mean injection velocity) between 1 and 5. The gel fibres were then washed in water either by manually dipping into a beaker (3 × 2 s) or by passing through an in-line washing bath (length 15–40 cm) at the specified take-up velocity. Sections of dipped fibres were hung to dry with a small tag of foil (20 mg), whilst the in-line washed fibres were supported across some guide pins to dry at constant strain in ambient conditions.

Cross-linked fibres were prepared by dipping the gel fibres in 0.1 M sodium succinate (98%, Sigma-Aldrich) for 2 × 15 s, followed by hanging to dry with a tag of foil (20 mg) in ambient conditions.

### Polarised optical microscopy

Optical microscopy of dried INT fibres and INT solutions was conducted using a Leica DM2500 optical microscope equipped with a Basler PowerPack Ace 2.3 MP camera in transmission illumination with a 100 W halogen lamp and Leica HiPlan objectives. Polarised optical microscopy was conducted using a transmission polariser and integrated analyser. For additional colour contrast, 560 nm retardation film (Edmund Optics WP560 retarder film) was positioned between the polariser and condenser lens at 45° to the polariser and analyser directions.

*In situ* polarised optical microscopy was conducted using a variable magnification digital microscope with a polarising filter (Dino-lite AM4113ZTL) and a lightbox with adjustable polariser (Dino-lite BL-ZW1).

### Single filament tensile testing

INT fibre samples were tested following BS ISO 11566:1996. Fibre samples were mounted onto card frames with a gauge length of 15 mm and the ends were fixed with epoxy adhesive (Araldite Rapid, Huntsman Advanced Materials Ltd.). The tensile tests were conducted on a TST350 tensile stress tester (Linkam Scientific Instruments Ltd.) with a 2 N load cell and a crosshead speed of 1 mm min^−1^ (strain rate 1.1 × 10^−3^ s^−1^). The cross sectional area of each sample was determined using transmission optical microscopy. Each tensile specimen was imaged over its full gauge length, with the diameter of the specimen taken as the mean of the apparent width. The cross sectional area was then calculated from this diameter, assuming a cylindrical fibre. If the fibre cross-section is irregular, this method will tend to overestimate the area, and hence underestimate the strength and modulus. Linear density was, therefore, calculated to evaluate tenacity, using the mass supply rate of imogolite (see ESI Note 1†). The similar trends in strength and tenacity, and the reasonable implied packing fractions (see below), lend confidence to the estimated cross-sections, although the actual moduli, and especially strengths, may be higher than reported here.

### X-ray scattering (XRS)

Small-Angle X-ray Scattering experiments on dilute INT suspensions were carried out at the SWING beamline of the SOLEIL synchrotron (Saint-Aubin, France) at a beam energy of 12 keV. Two-dimensional patterns were recorded using an Eiger 4M detector (Dectris Ltd., Switzerland) with pixel size 75 μm, placed in a vacuum tunnel at a sample-to-detector distance of around 6 m. Suspensions of INT were held in borosilicate tubes (diameter 1 mm, WJMGlas/Müller GmbH, DE) that were flame-sealed and stored vertically prior to experiments.

Wide-angle X-ray scattering experiments on INT fibres were conducted using a rotating anode (RU H3R, Rigaku Corporation) using Cu Kα radiation (*λ* = 0.154 nm) with multilayer W/Si monochromator. Short lengths of fibre or bundles of a few pre-aligned fibres were mounted on cardboard frames, placed perpendicular to the incident X-ray beam and exposed for 2 hours on a MAR345 two-dimensional detector (marXperts GmbH) with a 150 μm pixel size and 300 mm sample-detector distance. Extraction of the scattered intensity as a function of scattering vector and azimuthal angle was performed with home-developed software.

## Results and discussion

### Rheological behaviour and flow alignment of INT solutions

Similarly to other rigid rod dispersions, aqueous solutions of imogolite nanotubes are highly shear thinning due to reorientation of the nanotubes in the flow direction^21,22^ Capillary rheometry was used to study solutions with INT concentrations varying from 1 to 11 mg mL^−1^ (volume fraction, *φ*, 0.037% to 0.41%, based on a mean INT density^13^ of 2.7 g cm^−3^) which represents the range of concentrations from isotropic to fully liquid crystalline solutions (Fig. S1†). Whilst at 11 mg mL^−1^ the solution is nematic, at 5.5 mg mL^−1^ (*φ* = 0.2%) a columnar phase forms, as confirmed with small angle X-ray scattering (Fig. S2†). All the INT solutions used in this study were found to be shear thinning. As shown recently,^23^ the viscosity of INT suspensions can be reproduced using an extended hard sphere approach based on Quemada's rheological model^24,25^ which gives,
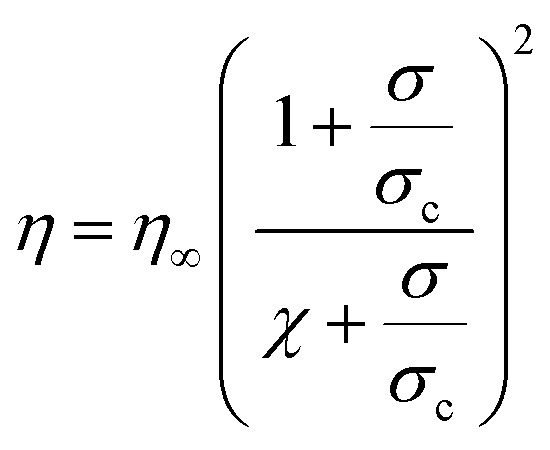
where *η* is the shear viscosity, *η*_∞_ is the viscosity under infinite shear stress, *σ* is the shear stress and *σ*_c_ is a critical shear stress, which corresponds to the point where the hydrodynamic effects are comparable to Brownian motion and other interactions. The parameter *χ* (for which −1 ≤ *χ* ≤ 1) reflects the state of the suspension. If the viscosity remains finite at zero shear, *χ* is positive and the zero shear viscosity is given as *η*_0_ = *η*_∞_/*χ*^2^. Suspensions which exhibit a yield stress at rest have a negative *χ* and the yield stress is given by *σ*_*y*_ = −*χσ*_c_. The flow curves can be well-reproduced using the three parameters of this model, *η*_∞_, *σ*_c_ and *χ*. For all of the concentrations studied here, the *χ* parameter is positive indicating that the suspensions remain fluid within this studied shear range; however, the value of *χ* reduces as the mass loading increases indicating a transition towards a more elastic network (Table S1†).

**Fig. 1:**
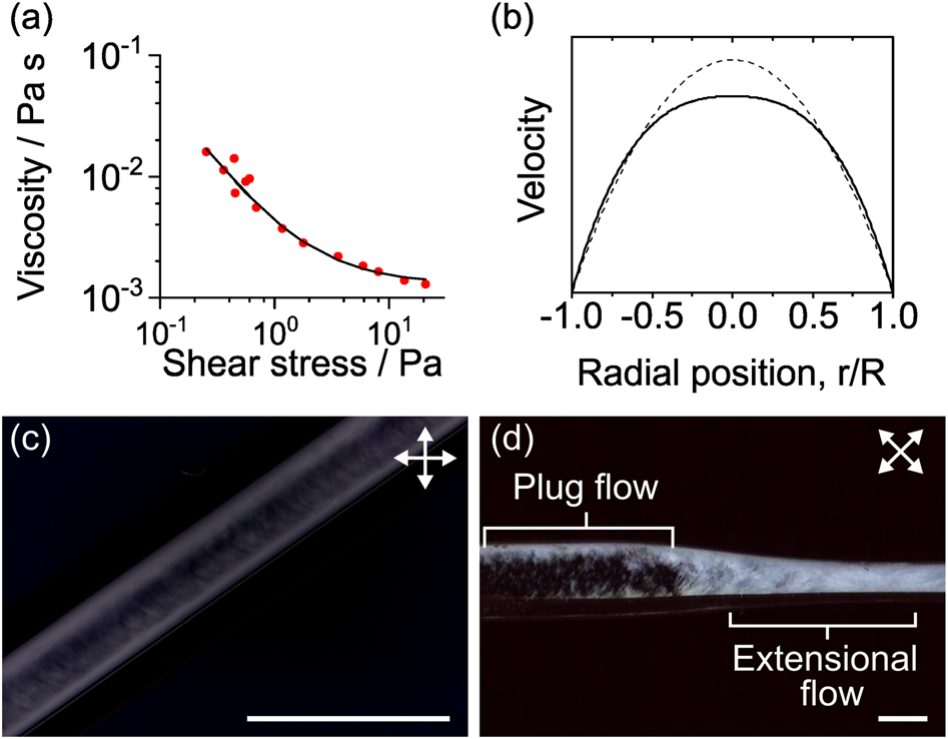
(a) Viscosity of 11 mg mL^−1^ solution of INTs in water. (b) Predicted velocity profile of INT solution flowing in a pipe (solid line) compared to a Newtonian fluid (dashed line). (c, d) Polarised optical micrographs of INT solutions flowing through (c) cylindrical and (d) tapered channels. White arrows indicate the polariser and analyser directions. Scale bars 1 mm.

The highest concentration, 11 mg mL^−1^, solution fits the rheological model with a *χ* parameter of 0.09 and critical shear stress *σ*_c_ of 0.99 Pa (Fig. 1a). The shear stress for flow in a cylindrical pipe can be given as,
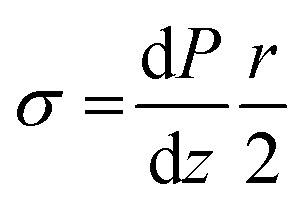
where d*P*/d*z* is the pressure gradient and *r* is the radial position within the flow. The velocity profile, *u*(*r*), can then be estimated by integrating the predicted shear strain rate, *γ̇* = *η*/*σ* = d*u*/d*r*, with respect to *r* and applying the boundary condition of zero velocity at the pipe edges, *u*(*R*) = 0 (*i.e.* assuming no wall slip). Compared to a Newtonian fluid, the velocity profile of this shear thinning solution is flattened with extensive shear occurring near the pipe walls and comparably little shear in the core (Fig. 1b). As previously seen in both INT and CNT suspensions, both the viscosity and the degree of shear thinning increased with volume fraction of INTs in the solution (Fig. S3†).^26,27^ The liquid crystalline 11 mg mL^−1^ INT solution was selected for further experiments in this work as the higher mass loading is expected to lead to larger and more easily handleable fibres.

The alignment of INTs flowing in cylindrical capillaries was visualised *via* polarised optical microscopy (POM). When the analyser and polariser are crossed at 45° to the capillary axis, bright birefringent regions of aligned INTs can be seen at capillary edges whilst a disordered core can be seen to translate along the centre of the channel with minimal shear (Fig. 1c and ESI Video†). It is known that the alignment of colloidal rods is significantly more efficient in extensional flow than shear flows.^28^ In order to achieve better INT alignment within the dope, a tapered channel was implemented. Tapered channels lead to a complex blend of shear and extensional flows as the fluid is sheared by the capillary walls and accelerated axially due to the deceasing channel cross-section but constant volumetric flow rate along the capillary length. In the parallel section before the taper, typical plug flow can be seen. However, at the start of the taper, extensional flow commences, and deformation is visible throughout the capillary section. The whole of the INT solution, including the core, is visualised as a bright birefringent region indicating its alignment by the extensional flow in the taper. As opposed to the cylindrical channel, the degree of alignment at the end of the taper is visibly homogeneous across the cross-section (Fig. 1d).

**Fig. 2:**
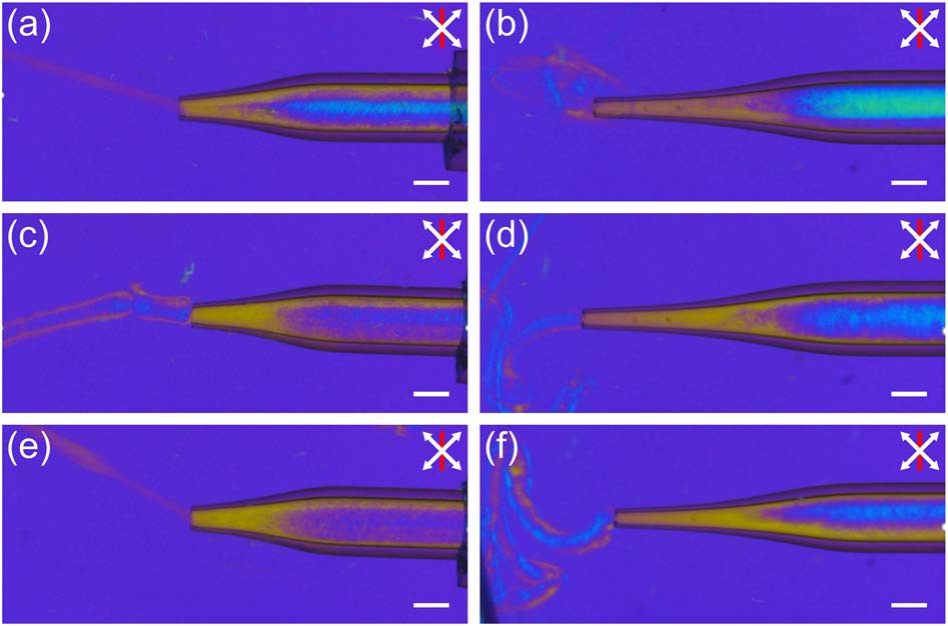
Polarised optical micrographs of INT solutions flowing freely through tapered glass nozzles at (a, b) 1, (c, d) 5 and (e, f) 10 mL h^−1^ into a static bath of 0.1 M sodium succinate without drawing. White arrows indicate the polariser and analyser directions, and red lines indicate the slow axis direction of a 560 nm full-wave retardation plate. Nozzles have similar taper semi-angles (8°) but different lengths (4 and 7 mm). Scale bars 1 mm.

The flow alignment was confirmed by inserting a full-wave *λ* plate in the optical path with the slow axis perpendicular to the capillary axis. This arrangement leads to colour contrast in the optical micrographs where isotropic material is magenta pink, regions with INTs aligned along the capillary axis are yellow and regions with INTs aligned perpendicular to the capillary are blue. Extensional flow alignment was found to be effective with two capillaries with different taper lengths (taper semi-angle ∼8°, taper lengths 4 and 7 mm) at volumetric flow rates from 1 to 10 mL h^−1^ (Fig. 2). INT alignment can be seen to occur within the taper region in all cases, although at higher flow rates the alignment occurs over a shorter channel length.

### Fibre spinning and drawing

**Fig. 3:**
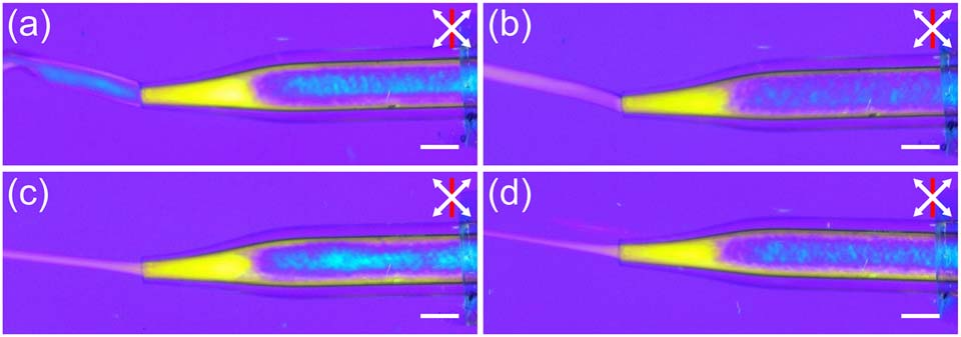
*In situ* polarised optical micrographs of INT fibre spinning with (a) no draw applied and (b–d) a draw ratio of (b) 1, (c) 3 and (d) 5. White arrows indicate the polariser and analyser directions, and red lines indicate the slow axis direction of a 560 nm full-wave retardation plate. Scale bars 1 mm.

In order to spin fibres, the aligned INT spin dopes were injected into a stationary aqueous CaCl_2_ coagulation bath and then collected on a take-up wheel with a draw ratio varying between 1 and 5. Without any applied take-up process, the proto-fibre decelerates rapidly upon entering the coagulation bath due to both die swell and frictional losses on entering the static bath. This deceleration leads to rapid misalignment of the spin dope, visible as a clear colour change in the POM images (Fig. 3a). Applying draw *via* the take-up roller prevents deceleration and maintains a mixed viscous and elastic stress in the proto-fibre during coagulation which preserves the alignment of the INTs (Fig. 3b–d). Take-up at higher draw ratios leads to greater deformation of the proto-fibres (Fig. 3) leading them to become significantly thinner, without a significant change in colour (retardation), indicating densification.

In order to assess the effect of draw ratio (DR) on fibre properties, fibres were spun with draw ratios of 1, 2, 3, 4 and 5. The gel fibres were washed in deionised (DI) water to remove residual CaCl_2_ by manually dipping the fibres two times for 3 s. The washed fibres were hung to dry with a small weight (∼20 mg) in ambient conditions. The resulting water-washed (WW) fibres were imaged using transmission optical microscopy and the mechanical properties were measured using a single filament tensile test at controlled humidity.

**Fig. 4:**
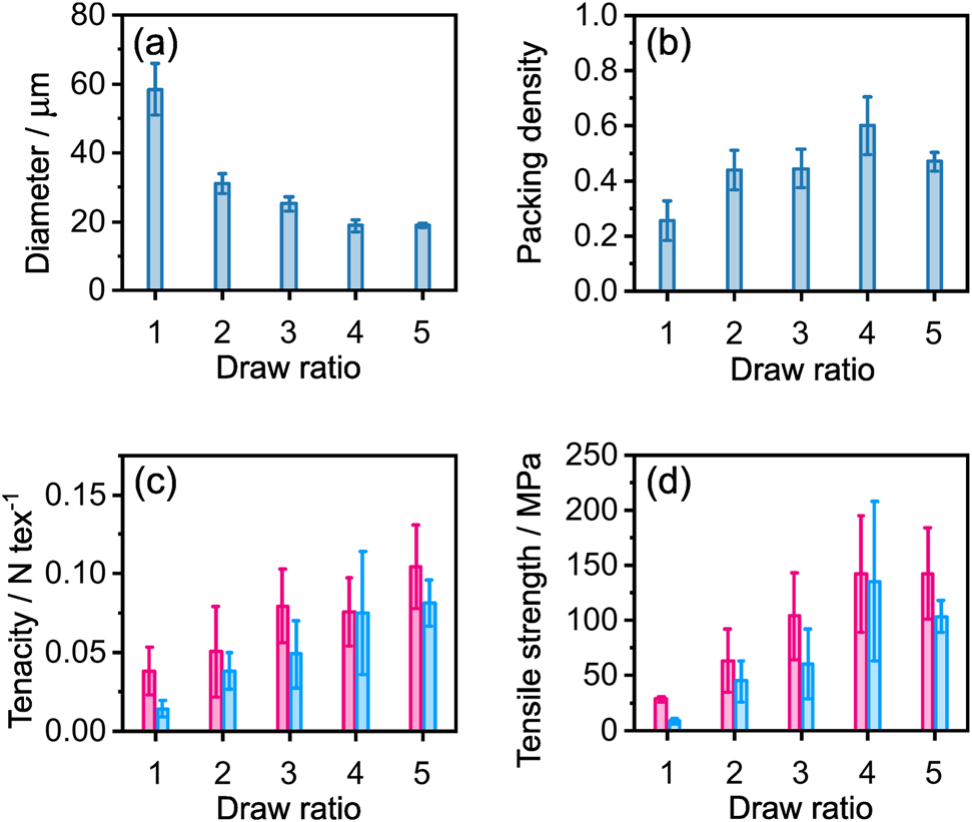
(a) Diameter, (b) packing density, (c) tenacity and (d) tensile strength of INT fibres prepared by coagulation in CaCl_2_ with a tapered spinneret at varying draw ratio followed by washing in DI water (3 × 2 s). Magenta bars indicate samples tested at 10% RH, cyan bars for samples tested at 40% RH. Error bars show ±1 standard deviation.

As expected, the cross-sectional areas of the dry fibres decreased with draw ratio (Fig. 4a). However, in addition, the INT packing density, determined as the volume of INTs divided by the fibre volume (ESI Note 1†), tended to increase across the series (Fig. 4b). For DR 1, the packing density was estimated to be 25%, rising to 60% at DR 4, although then decreasing to 47% at DR 5. This trend is reflected in the tensile data with the tenacities increasing with draw ratio at both 10 and 40% RH (Fig. 4c). Since tenacity depends on linear density, not cross-section, the increase indicates more efficient use of the INTs, due to better inter-tube load transfer at higher packing density. The relative increase in strength (Fig. 4d) is even greater as the better packing density also reduces the fibre cross-section (Fig. 4d).

Qualitatively, the fibres prepared at DR 1 appeared wetter than the other fibre samples and glistened under standard lab illumination, indicating that the CaCl_2_ coagulant was not fully removed during washing. For the consistent washing treatment used here, the efficiency of the washing depends upon the diameter of the gel fibre; a longer time period is required to allow salt to diffuse from the larger fibres. The improved mechanical properties in the fibres with the higher draw ratio can, therefore, be largely attributed to the more efficient removal of the coagulation salt which increases both packing density and the associated number of nanotube contacts within the fibre.

### Washing strategies for microstructural control

Since fibres collected directly from the CaCl_2_ bath cannot fully dry under ambient conditions, due to the presence of the hygroscopic salt within them (Fig. S4†), a water wash is needed to remove excess salt and enable densification. However, this water washing leads to swelling and solubilisation of the INT gel fibres, causing them to become significantly weaker and more challenging to handle, over time. After 1 minute, it was not possible to remove the fibre from the washing bath in a continuous piece and after 10 minutes the fibre almost completely dissolved. Changes to the gel fibre during washing were observed using *in situ* POM (Fig. S5†). After just 10 s of immersion, the gel fibres clearly swell compared to their initial state, and this swelling increased over time with fibrillation visibly occurring after 5 minutes. During the washing process, the intensity of the birefringence colours decreased due to both the reduced concentration of the swollen gel and likely a reduction in alignment.

**Fig. 5:**
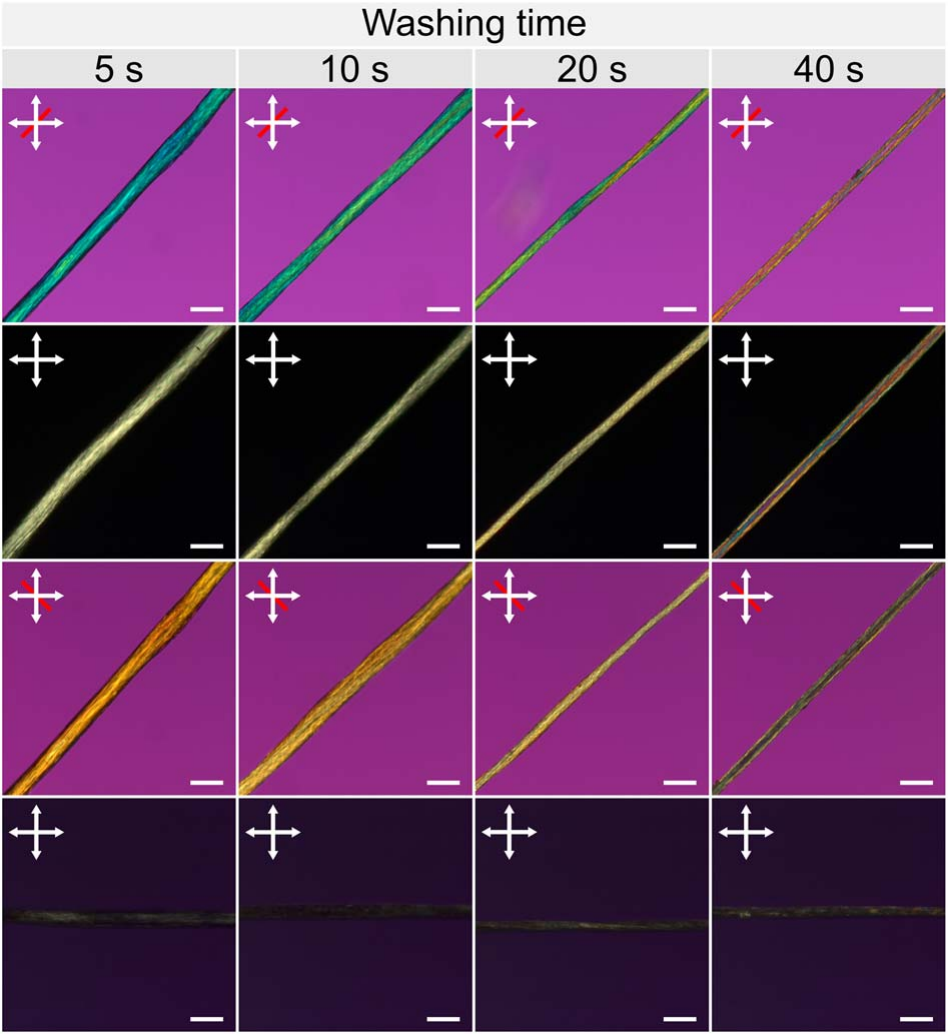
Polarised optical micrographs of dry INT fibres produced with DR 2 and washed by submersion in water for varying periods of time. White arrows indicate the polariser and analyser directions, and red lines indicate the slow axis direction of a 560 nm full-wave retardation plate. Scale bars 100 μm.

POM was then used to observe the evolution of the microstructure of the final, dried, INT fibres as a function of washing time (Fig. 5). Fibres were spun at two different draw ratios (2 and 4), dipped into water for 5, 10, 20 or 40 s, and then dried under tension (∼20 mg). Fibres washed for 5 s never completely dry under ambient conditions and had a larger diameter and glistening wet appearance under the microscope. After a critical washing time of ∼20 s for DR 2 (Fig. 5), the fibre birefringence changed significantly. With the fibre positioned at 45° to the crossed polarisers, the total retardation increased from ∼200 nm at short washing times to ∼500 nm after longer washes. This higher retardation likely arises due to the improved densification after long washes, where a greater quantity of INTs are packed into the light path for the thinner, denser fibres. At the same time, after longer washes, a significantly stronger birefringent signal can be seen when the fibre is aligned parallel to the analyser, showing a greater degree of misalignment. These changes indicate that after longer washing times, the dried fibres have an increased INT packing density but contain more bundles which are misaligned relative to the fibre axis. The simultaneous increase in packing density and decrease in alignment may seem counter-intuitive when considering simple packing of rigid rods. However, in this case, the structural rearrangements occur alongside the removal of highly hygroscopic salt from the fibres. The more aligned fibres from shorter washing times are swollen with adsorbed water, the deliquescence of which can be visualised, which leads to their lower packing density.

Having identified that washing conditions lead to significant changes in INT fibre morphology, an inline washing bath was introduced to the wet spinning rig to provide greater experimental consistency during continuous spinning. After initial draw and take-up from the coagulation bath the gel fibre was passed *via* guide rods through DI water baths of length 15, 30 and 40 cm corresponding to washing times varying between 8 and 23 s for DR 2, and 4 and 11.5 s for DR 4. It was not possible to use longer washing baths due to weakening of the gel fibre leading to frequent filament breakage on the continuous spinning line.

As before, structural changes in the dried fibres were observed under POM with increased retardation at longer washing times. The POM observations again indicate the occurrence of two key processes: diffusion of CaCl_2_ out of the gel fibre and structural rearrangement of the INTs due to partial solubilisation. The significance of these processes may be compared by considering the characteristic time scales over which they occur.

The characteristic diffusion time required for calcium and chloride ions to be washed from the gel fibre can be estimated by considering diffusion of the salt as the random walk of a Brownian particle radially (perpendicular) to the fibre axis. The root mean squared displacement in this random walk is given by,1
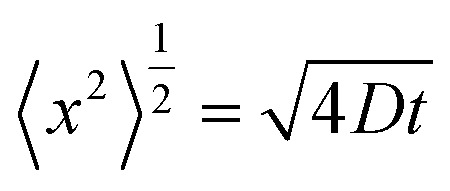
where *D* is the diffusion coefficient and *t* the diffusion time.^29^ The characteristic time can be estimated as that required for a root mean squared displacement equal to the initial gel fibre diameter (319 and 226 μm for DR 2 and DR 4 respectively). Using a diffusion coefficient for CaCl_2_ in aqueous solutions^30^ of 1.2 × 10^−9^ m^2^ s^−1^ yields characteristic diffusion times of 21 and 11 s for DR 2 and 4, respectively.

The characteristic timescale for structural rearrangement can be estimated as *τ*_r_ ∼ 1/*D*_r_ where *D*_r_ is the rotational diffusion coefficient for the INTs in the fibre. By modelling the INTs in the gel fibre as a semi-dilute suspension of thin rigid rods with a concentration equivalent to the spinning dope, the rotational diffusion coefficient can be determined by following Doi^31^ as,2
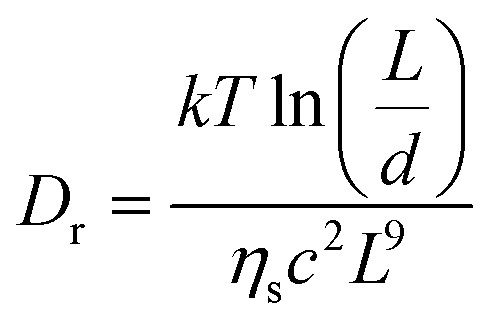
where *kT* is the Boltzmann constant multiplied by temperature, *L* is the INT length, *d* is the INT diameter, *η*_s_ is the solvent viscosity and *c* is the number concentration of rods. This estimate is extremely dependent upon the INT length distribution, which is broad and bimodal.^13^ Considering characteristic INT lengths of 600 and 250 nm gives a rotational relaxation times, *τ*_r_, around 120 and 0.3 s, respectively. Hence, short INTs will reorient more significantly than longer ones, while longer INTs are likely to be particularly important to maintain the structural integrity of the fibre during washing. In fact, the solubilisation of the INTs which enables structural rearrangement is only expected once a sufficient amount of the coagulating salt has been removed. The onset to the structural relaxation should therefore vary with radial location in the fibre. Despite these complexities, it is clear that the removal of residual salt and structural rearrangement occur over similar timescales. For these reasons, it is challenging to fully decouple the salt extraction and INT relaxation, experimentally.

**Fig. 6:**
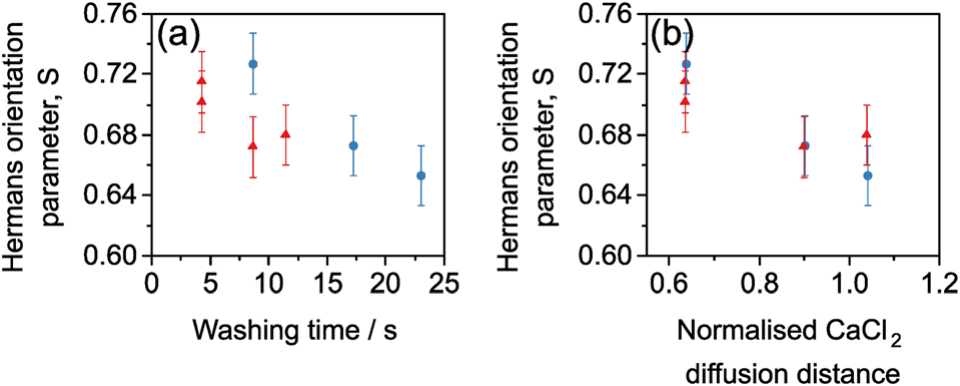
Hermans orientation parameter for INT fibres spun at (blue circles) DR 2 and (red triangles) DR 4 as a function of (a) the washing time and (b) the CaCl_2_ diffusion distance divided by the initial gel fibre diameter.

Wide angle X-ray scattering was used to characterise, further, the relationship between draw ratio, washing and the INT alignment. The fibre scattering patterns show the typical features of the DW Ge-INTs used in this work with large oscillations visible on the equatorial line at scattering vectors of 2.6, 5.4 and 7.3 nm^−1^ (Fig. S6†). The alignment was characterised by fitting the angular dependence of the scattered intensity at 2.6 nm^−1^ to a Lorentzian function. As previously described,^13,32,33^ the reciprocal space Lorentzian of the X-ray scattering pattern arises from a direct space orientational distribution function (ODF) of a Lorentzian to the power 1.5, with a half-width-at-half-maximum (HWHM), *w*_d_ = 0.775 *w*_r_, where *w*_r_ is the HWHM of the Lorentzian fit to the reciprocal space diffraction data. The Hermans' order parameter, *S*, is then calculated from the ODF as3
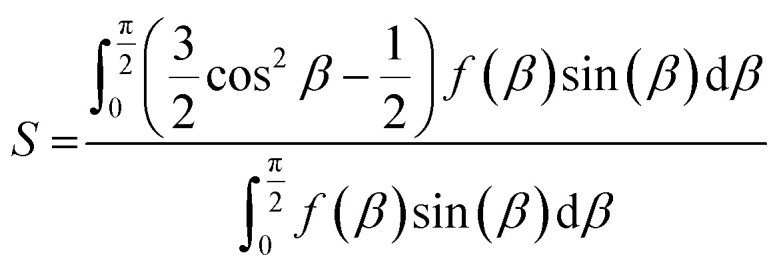
where *f*(*β*) is the Lorentzian function to the power 1.5 as a function of the polar angle *β* and the denominator is used here to normalise the ODF to 1. For both draw ratios, the order parameter, *S*, decreased monotonically with washing time, confirming the misaligning effect of washing in DI water (Fig. 6a). However, the thinner DR 4 gel fibres misalign more rapidly during washing than the thicker DR 2 gel fibres. Quantifying the washing step, instead, as the root mean squared diffusion distance for CaCl_2_ during the washing time, divided by the initial gel fibre diameter, collapses the data to a master curve where the INT alignment is equivalent for DR 2 and DR 4 (Fig. 6b). The degree of structural rearrangement in the fibres is therefore shown to be intrinsically linked to the CaCl_2_ diffusion. Interestingly, increasing draw ratio reduces the length of time in the washing bath (*t* ∝ 1/DR) and the gel fibre diameter (*d* ∝ √DR) in such a way that it does not impact the effective degree of washing (time needed for diffusion ∝ *d*^2^ ∝ (√DR)^2^ ∝ DR; time available for washing *t* ∝ 1/DR). Instead, the degree of washing is controlled by the extrusion rate and the length of the washing bath such that:4
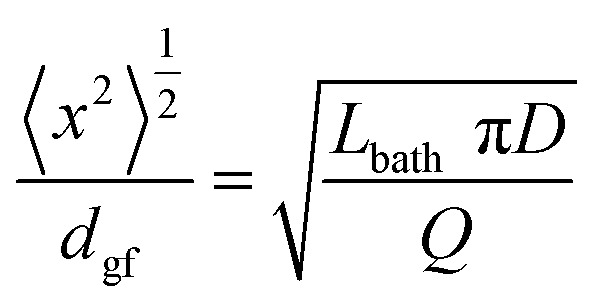
where *d*_gf_ is the gel fibre diameter as it enters the washing bath, *L*_bath_ is the length of the washing bath, *D* is the CaCl_2_ diffusion coefficient and *Q* is the volumetric flow rate of spinning dope (ESI Note 2†). The POM (Fig. 3) indicates that pre-alignment of the INTs in the proto gel fibre occurs mostly in the taper, with low dependence on the draw ratio. Given the low INT volume fraction (0.41%) in the dope, the extensional deformation of the gel fibre by drawing is, most likely, dominated by longitudinal slip rather than an affine deformation that reorients the nanotubes. This argument predicts a much stronger dependence of mechanical properties on washing bath length than on draw ratio.

### Mechanical properties as a function of processing conditions

**Fig. 7:**
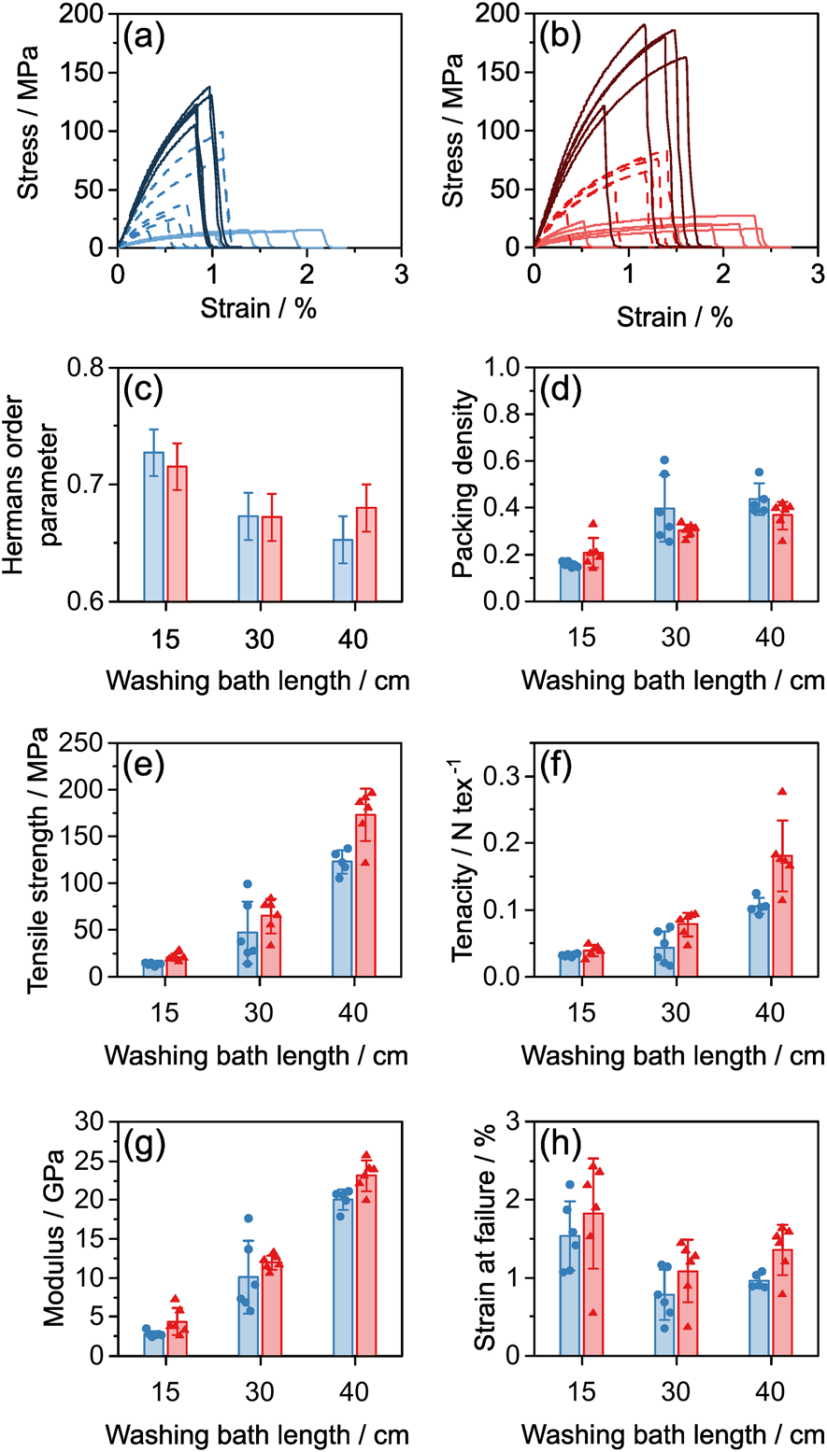
(a, b) Stress strain curves for INT fibres spun at (a) DR 2, (b) DR 4 using a tapered spinneret. Dark solid lines 40 cm washing bath, medium dashed lines 30 cm washing bath, light solid lines 15 cm washing bath. (c–h) Structural and mechanical parameters for (blue) DR 2 fibres and (red) DR 4 fibres with varying washing bath lengths. Error bars indicate 1 standard deviation.

The tensile mechanical properties of these fibres show that longer washing baths resulted in significantly stiffer and stronger fibres which tended to have smaller strains to failure (Fig. 7a and b). For example, for DR 4, the tensile strength increased from 20 ± 4 to 170 ± 30 MPa, the modulus increased from 4.3 ± 1.7 to 23 ± 2 GPa and the strain-at-failure decreased from 1.8 ± 0.7 to 1.3 ± 0.3% as the washing bath length increased from 15 to 40 cm. The packing density, alignment, stiffness, strength, tenacity and strain-at-failure all follow the same trends for both draw ratios (Fig. 7c–h), in agreement with the prediction that washing bath length (eqn (4)) is the critical process factor affecting the fibre properties.

As washing bath length increases, the INT orientation reduces (Fig. 7c), in agreement with previous data that less aligned INT fibres are stiffer^13^ but now identifies that both the removal of CaCl_2_ and structural rearrangements during washing are contributing process factors. As the bath length increased from 15 cm to 30 cm, packing density also increased from ∼20% to ∼40%. Whilst it is difficult to separate the effects of packing density and orientation, increased strength and stiffness can be attributed to the formation of an interlocked network of the misaligned INTs. Compared to other nanomaterial fibres such as CNT fibres, the INTs used here are relatively short (mostly 200–600 nm) and are extremely straight and rigid, as can be seen in previous TEM images.^13^ As such, the typical model of nanotube stress transfer due to van der Waals' interactions within locally parallel bundles is less relevant within these fibres. The rigid nanotubes cannot easily bend to form contacts, or parallel bundles, and hence they have a low contact area and poor stress transfer. In the more aligned fibres, ductile sliding can occur easily without arrest which results in the lower strengths and higher strains to failure seen at short washing lengths. In contrast, less aligned structures may transfer stress through a network of jammed frictional rods. The formation of these jammed networks occurs when the number of independent contacts on each rod reaches a critical value of around ten.^34^ Increasing packing density and increasing misalignment both help to create additional independent contacts between INTs, leading to the formation of a percolating network which can transfer tensile loads. Similar stress transfer networks have been formed by creating branched aramid nanofibers which have greater interconnectivity.^35^ These data match observations of shear thickening in suspensions of colloidal rods where sudden increases in shear stress are associated with the formation of a disordered percolating network, which has greater frictional interactions in this shear jammed state.^36^ Whilst there is a lack of models directly studying the effect of alignment on the mechanical properties of rod networks, recent progress has been made comparing the elasticity of rod and L-shaped particles in randomly oriented networks^37,38^ and it is expected that these approaches may be extended to explore other orientation distribution functions. A key existing result is that rod networks are always shear thinning, implying that as the alignment of the network increases during shear, its elasticity decreases in direct agreement with the argument presented above. In comparison, the constraints of the surrounding network on L-shaped particles can force them to bend leading to a shear thickening behaviour. Following this model, it may be beneficial to explore synthesis routes which create INTs with bends or kinks that further constrain the network and contribute to stress transfer.

INT fibres were also spun using a cylindrical capillary as the spinneret, showing properties in similar ranges, but with no clear trends within the large experimental scatter (Fig. S7†). Given the misalignment in the core of cylindrical nozzles (Fig. 1c), there fibres were expected to have locally variable alignment, both radially, and longitudinally due to the retained domain structure of the spinning dope. The variable structure gives rise to variable properties. In comparison, the more homogeneous gel fibre extruded from the tapered spinneret results in significantly more consistent properties, again emphasising the advantages of using the extensional flow field of the tapered spinneret to align the nematic spin dope.

### Gel cross-linking of INT fibres

The mechanical properties of the water-washed (WW) INT fibres have been shown to be dependent upon alignment and packing fraction, as the inter-nanotube contacts are highly mobile and require jamming constraints for effective stress transfer. Logically, it should be possible to create stiffer networks of more aligned INTs by cross-linking to increase the inter-nanotube shear strength.

Inspiration for cross-linking was taken from sodium alginate gels which can be physically cross-linked with divalent cations such as calcium to create robust networks.^39^ A potential beneficial side-effect is that successful gels may be water stable, enabling a greater range of washing conditions. By analogy to alginates, it was hypothesised that inorganic salt solutions with multivalent anions would effectively gel INT solutions, assuming that ionic cross-links form between the positively-charged outer surfaces of the INTs. Experimentally, INT dope was injected by hand through a 21 g needle into a small vial (30 mL) of coagulant solution. The resulting gel fibres were collected and placed in a Petri dish of deionised water and the condition of the gel fibre was qualitatively assessed at various intervals by gently handling the fibre with tweezers and noting it as either robust or fragile (Table 1).

**Table 1:** Stability of INT fibres prepared with various coagulants after washing in water. At each washing time the fibres were recorded as either robust (✓) or fragile (×)

Coagulant	Washing time/minutes				
	0.5	1	5	10	30
Sodium chloride	×				
Calcium chloride	✓	×			
Sodium citrate	✓	✓	✓	✓	✓
Sodium succinate	✓	✓	✓	✓	✓
Sodium tripolyphosphate	✓	✓	✓	✓	✓
Sodium sulphate	✓	✓	✓	✓	✓

Fibres coagulated in salts with monovalent anions (NaCl and CaCl_2_) rapidly weakened when soaked in water irrespective of the cation valency. However, fibres coagulated in salts with multivalent anions remained robust even with prolonged soaking in water (over 30 minutes) indicating the success of the ionic cross-linking strategy. However, trials seeking to draw fibres spun into cross-linking coagulants were unsuccessful, across a broad range of coagulant concentrations from 0.001 to 1 M. Instead of deforming smoothly at the spinneret nozzle, the proto-fibres fractured uncontrollably. This behaviour is likely due to the formation of a rigid network on the surface of the proto-fibre which cannot stably draw under the elongation. The resulting increase in elastic stress within the fibre results in its abrupt failure.

Gel cross-linked fibres (GCL) were instead prepared by initial spinning into a CaCl_2_ coagulation bath and then washing in a 0.1 M solution of sodium succinate (2 × 15 s). Compared to water washed fibres (WW), the GCL fibres' mechanical behaviour was significantly less sensitive to humidity, indicating that the cross-linking sites inhibit the moisture mediated sliding previously seen in pure INT fibres. However, the absolute mechanical properties of the GCL fibres were significantly worse than WW fibres, with a larger cross-sectional area, smaller tensile strength and smaller tenacity (and hence breaking force) for equivalent conditions (Fig. S8†).

The significantly larger cross-sectional area for GCL fibres results from poor densification of the gel fibres during drying. The formation of the rigid network apparently inhibits diametral shrinkage and prevents the formation of additional contact points between INTs, leading to a more porous structure which is less able to transfer stress. Importantly, the difference in strength between the GCL and WW fibres is not simply due to the different cross-sectional area, since there is also a reduction in the absolute breaking force and hence tenacity of the fibres.

This ionic cross-linking method can create robust aqueous gels resistant to redissolution, which may be useful in other contexts. However, these cross-linked gel fibres are not attractive as a route to improved, dense fibres. Instead, in order to improve humidity stability and yield potential improvements to fibre strength, cross-linking strategies should be targeted on dry, pre-densified fibres.

## Conclusions

The structure and properties of pure INT fibres are controlled *via* several process variables in wet spinning. The shear thinning rheology of INT solutions creates challenges during spinning as plug flow in cylindrical spinnerets leads to inhomogeneous alignment in the dope and inconsistent fibre properties. Tapered spinnerets, which induce extensional flow fields, ensure deformation throughout the spin dope resulting in significantly more aligned and homogeneous gel fibres and correspondingly more consistent fibre properties. Tapered spinnerets are widely used in commercial processes, but should be considered more often in new materials development labs, where cylindrical needles often dominate; *in situ* observations will help to optimise geometries to dopes with different rheologies.

Spinning pure fibres from short, rigid nanotubes is quite different to typical composite systems, in which a matrix transfers stress between the rigid elements. The properties of pure porous INT fibres depend strongly on both misalignment and packing fraction. It is proposed that the formation of jammed, mechanically percolating rod networks underpin the increase in strength and stiffness with decreasing INT alignment. Fibre microstructure can be controlled by washing the gel fibre in DI water. Longer washing times lead to the removal of hygroscopic salt, and improved densification, whilst at the same time allowing structural relaxation leading to a greater amount of off-axis INTs. These two processes are strongly coupled during the wet spinning of INT fibres and occur over similar time scales, making it challenging to independently investigate the effects. Similar washing and relaxation processes will occur in many (nanomaterial) systems, and may be better understood by *in situ* analysis.

INTs can be simply crosslinked using multivalent anions to form robust aqueous gels resistant to redissolution. In the context of structural fibres, cross-linking the wet proto-fibres locks in a swollen state, limiting consolidation and leading to poorer mechanical properties than unlinked fibres. However, in other contexts proposed for imogolite based materials, for example as sorbents or catalyst supports, the ability to create stable, porous, network structures may prove useful.

The best process identified for creating consistent, high strength INT fibres uses a tapered spinneret to achieve a homogeneous dope followed by drawing in a CaCl_2_ coagulant, that allows for deformation and then washing in DI water; the result is a pure, relatively dense, jammed network structure suitable for tensile stress transfer. Although cross-linking INT fibres in the gel state has not improved the mechanical properties due to challenges with densification, increasing the shear strength of the inter-nanotube contacts in the dense, dry fibres should lead to increased strength and stiffness. Possible methods may include cross-linking *via* dehydration reactions or infiltrating polymer matrices. The preference for tapered spinnerets and delaying any permanent cross-linking until after consolidation is likely relevant to other one dimensional nanomaterial fibres, including CNT and other nanorod systems. *In situ* analysis is a key tool to accelerate the understanding and development of coagulation spinning processes.

## Author contributions

Conceptualisation: JFM, MSPS; formal analysis: JFM, EP; investigation: JFM; software: PL; validation: PL; supervision: PL, MSPS; writing – original draft: JFM; writing – review and editing: EP, PL, MSPS.

## Conflicts of interest

There are no conflicts to declare.

## Supplementary Material

NA-005-D3NA00013C-s001
