# Supplementary material for: Wet spinning imogolite nanotube fibres: an in situ process study
Source: Nanoscale Adv. 2023 May 31;5(12):3376–85. doi: 10.1039/d3na00013c (PMC10263001; doi:10.1039/d3na00013c)
Supplement: NA-005-D3NA00013C-s001 [file NA-005-D3NA00013C-s001.pdf]

Electronic Supplementary Material (ESI) for Nanoscale Advances.
This journal is © The Royal Society of Chemistry 2023

Supporting Information

Wet spinning imogolite nanotube fibres: an in-situ process study

Joseph F. Moore,[a] Erwan Paineau,[b] Pascale Launois[b] and Milo S. P. Shaffer*[a,c]*

[a] Department of Materials, Imperial College London, Exhibition Road, SW7 2AZ, UK.

[b] Université Paris-Saclay, CNRS, Laboratoire de Physique des Solids, 91405 Orsay, FR

[c] Department of Chemistry, Imperial College London, Exhibition Road, SW7 2AZ, UK.

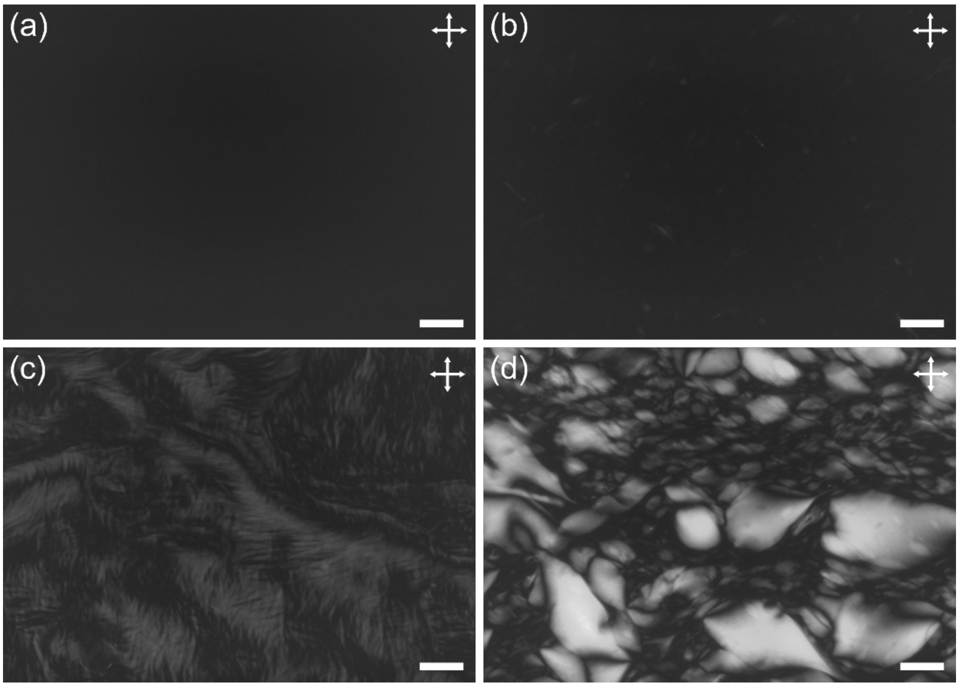

Fig. S1 Polarised optical micrographs of INT solutions with mass loading of (a) 1, (b) 3, (c) 5.5 and (d) 11 mg_{INT} mL^{-1}. Sampes were collected in rectuangular capillaries (thickness 200 µm) and allowed to relax for 1 month. White arrows indicate polariser and analyser directions. Scale bars 200 µm.

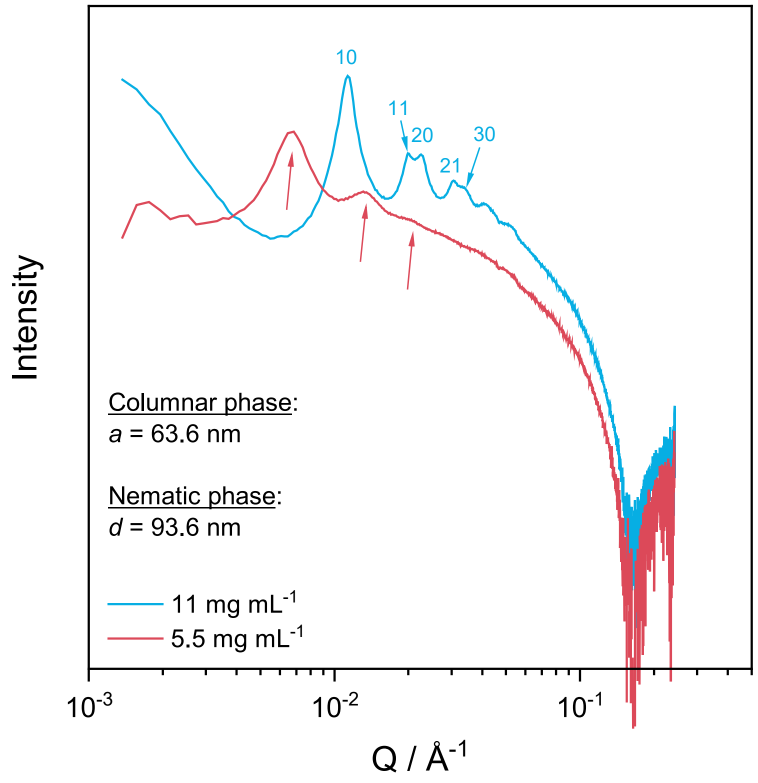

Fig.S2 SAXS intensity as a function of scattering modulus, Q for (blue) 11 mg mL-1 and (red) 5.5 mg mL-1 INT solutions with indexed scattering peaks for the columnar phase and corresponding lattice parameters, and typical INT separation distance for the nematic phase.

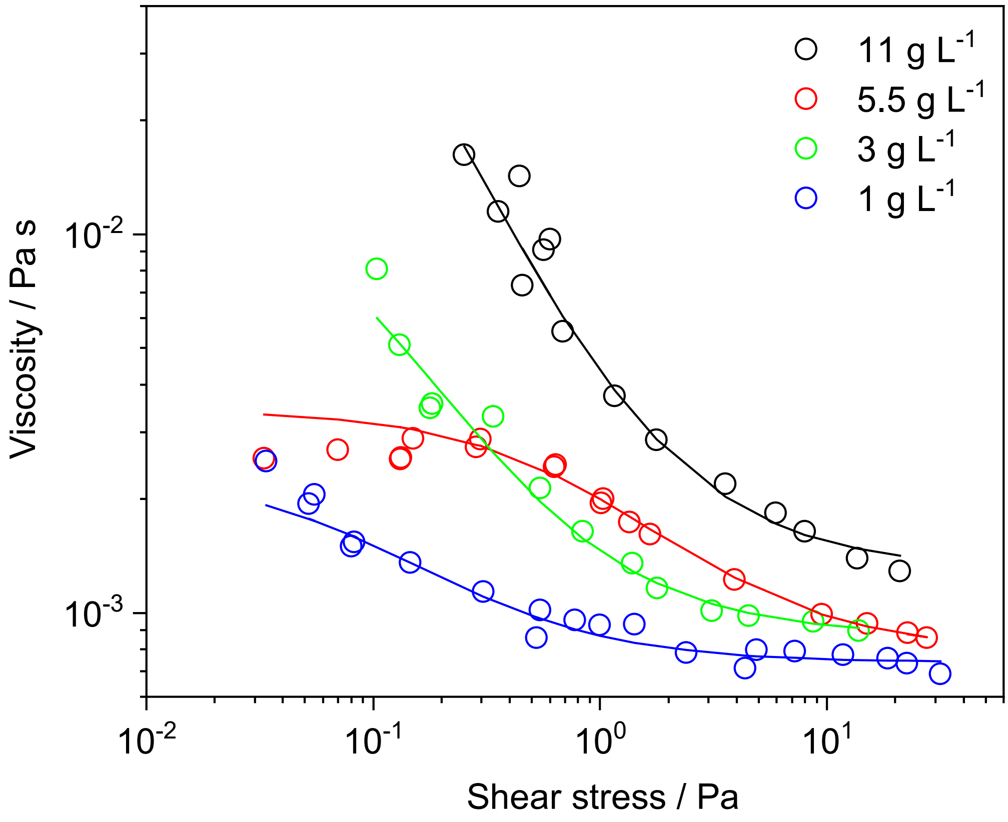

Fig. S3 Viscosity of INT solutions at varying INT mass loadings as determined by capillary rheometry. The deviation of the 5.5 mg mL^{-1} solution from the trend is likely due to the onset of liquid crystallinity as seen in Figure S1.

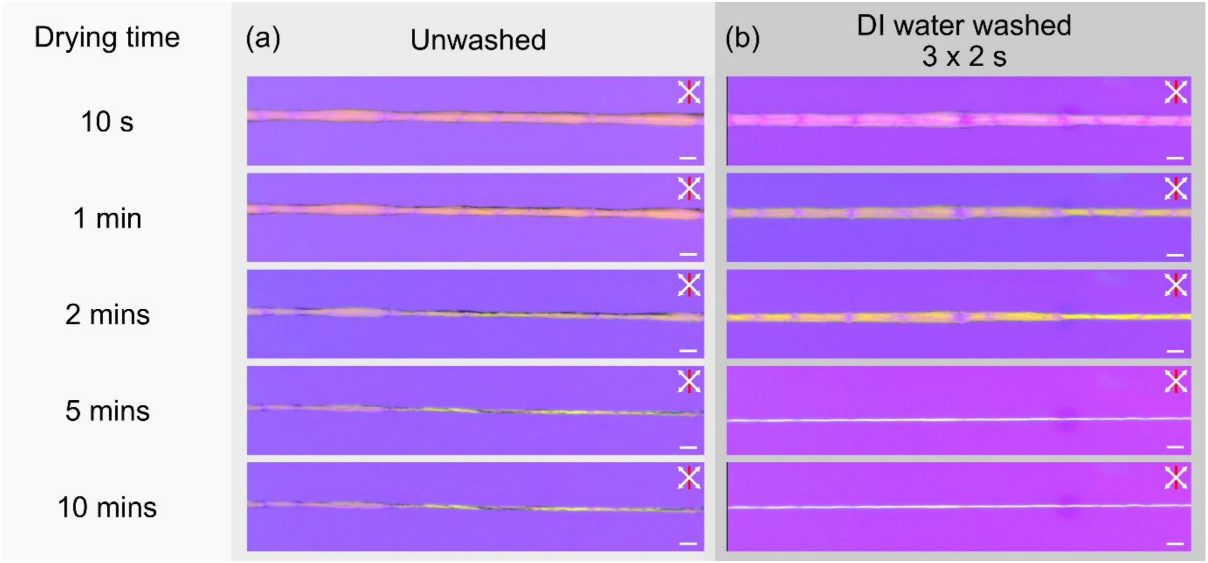

Fig. S4 *In-situ* polarised optical micrographs of INT fibres drying in ambient conditions (a) with no washing and (b) after washing by immersion in DI water for 3 x 2 s. White arrows indicate polariser and analyser directions and red lines indicate the slow axis direction of a 560 nm full-wave retardation plate. Scale bars 500 µm.

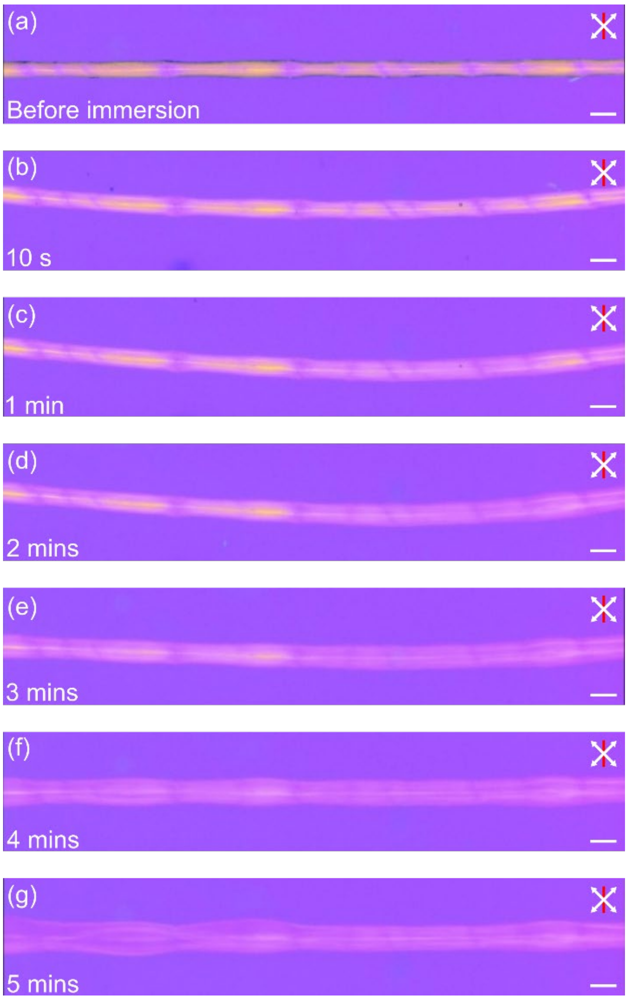

Fig. S5 Polarised optical micrographs of INT fibre spun at DR 2 during immersion in DI water. White arrows indicate polariser and analyser directions and red lines indicate the slow axis direction of a 560 nm full-wave retardation plate. Scale bars 500 µm.

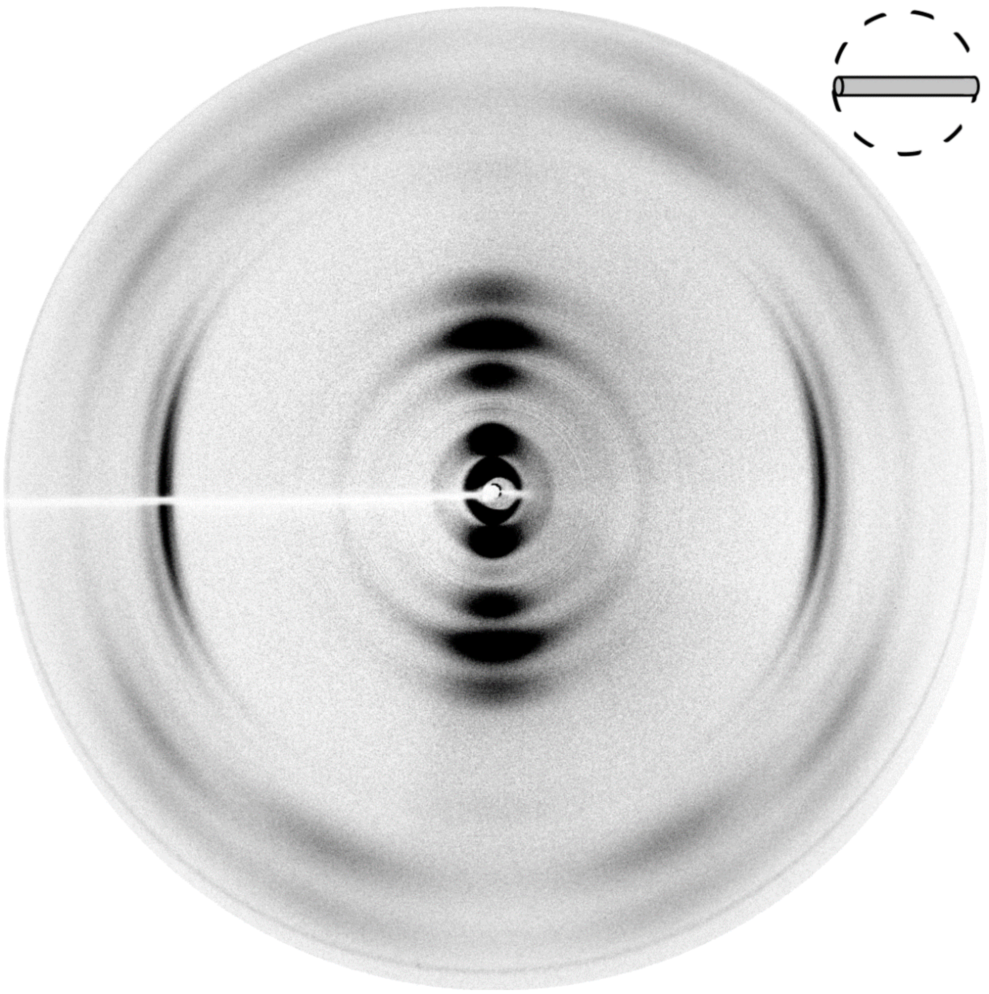

Fig. S6 Typical wide-angled X-ray scattering pattern of INT fibres produced in this work. The fibre is aligned with the horizontal direction as indicated in the (upper-right) schematic.

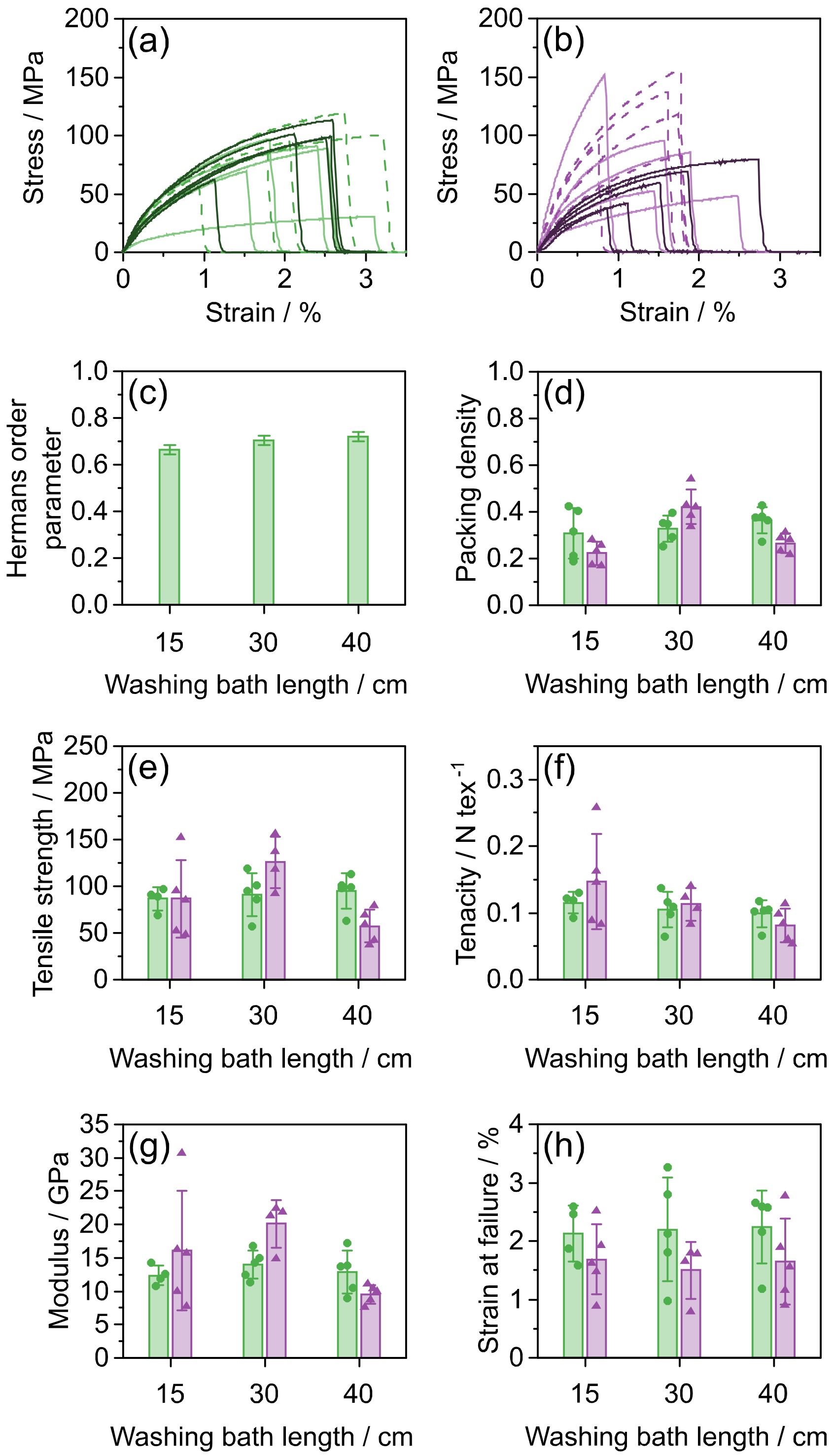

Fig. S7 (a,b) Stress strain curves for INT fibres spun at (a) DR 2, (b) DR 4 using a cylindrical spinneret. Dark solid lines 40 cm washing bath, medium dashed lines 30 cm washing bath, light solid lines 15 cm washing bath. (c-h) Structural and mechanical parameters for (green) DR 2 fibres and (purple) DR 4 fibres with varying washing bath lengths. Error bars indicate 1 standard deviation.

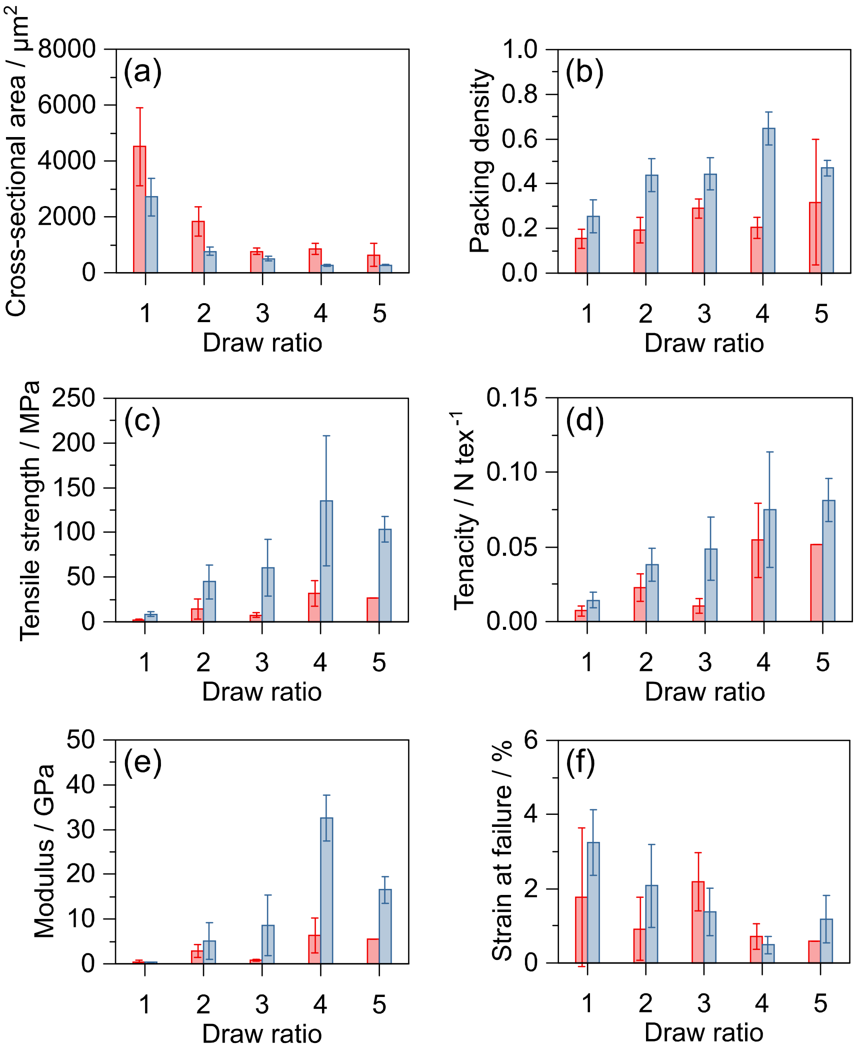

Fig. S8 (a,b) Structural and (c-f) mechanical properties of (red) gel cross-linked and (blue) water washed INT fibres tested at 40% RH. Error bars indicate 1 standard deviation.

Table S1 Fitting parameters for Quemada rheological model. Note that the values for 5.5 mg mL^{-1} may not follow the trend due to the onset of liquid crystallinity as shown in Fig. S1.

Solution mass loading / mg_{INT} mL^{-1}	χ	σ_c / Pa
1	0.56	0.21
3	0.22	0.41
5.5	0.47	2.47
11	0.09	0.99

Supplementary Note 1

A calculation of the packing density of the INT fibers has been previously derived in

https://doi.org/10.1021/acsami.1c00971 by the same authors; for clarity, the derivation is included here.

The packing density is estimated using the volumetric dope injection rate Q, the dope mass concentration c (mass of dry INTs per unit volume of the aqueous dope), the needle area A, the spin-draw ratio DR, the dry fiber diameter d, and the density of a DW Ge-INT ρ_{INT} . The density of a DW Ge-INT was calculated using the density of the nanotube walls (3.6 g cm^{-3}) and the outer diameter (4.34 nm), internal diameter (1.54 nm) and nanotube wall thickness (0.6 nm) to give ρ_{INT} = 2.7 g cm^{-3} as the mass of a nanotube divided by its envelope volume (i.e. the cylinder given by its outer diameter).

The volume of INTs injected in time τ, V_{INTs} is given by:

$$V_{INTs} = \frac{Q\,c\,\tau}{\rho_{INT}}$$

The volume of fiber produced in time τ, V_{fibre} is given by:

$$V_{fibre} = \frac{\pi\,d^2}{4} \times DR\,(\frac{Q\,\tau}{A})$$

The resulting packing density is given by:

$$Packing\ density = \frac{V_{INTs}}{V_{fibre}} = \frac{4\,c\,A}{\pi d^2 \times \rho_{INT} \times DR}$$

Similarly, the linear density, the mass of imogolite (M_{INT}) per unit length (L_{fibre}) is given by:

$$Linear\ density = \frac{M_{INTs}}{L_{fibre}} = \frac{Q\,c\,\tau}{Q\ \tau \frac{DR}{A}} = \frac{cA}{DR}$$

Supplementary Note 2

The root mean squared diffusion distance for $CaCl_2$ in the in-line washing bath, $\langle x^2 \rangle^{\frac{1}{2}}$, is given in equation (1) as,

$$\langle x^2 \rangle^{\frac{1}{2}} = \sqrt{4\,D\,t_{bath}}$$

where D is the diffusion coefficient and t_{bath} is the resisdence time in the washing bath.

The residence time in the washing bath is determined by the spinning conditions as the length of the bath divided by the take-up velocity such that,

$$t_{bath} = \frac{L_{bath}\,A_{nozz}}{Q\ \ DR}$$

where L_{bath} is the washing bath length, A_{nozz} is the spinneret nozzle area, Q is the volumetric flow rate of the spinning dope and DR is the draw ratio.

Therefore the root mean squared diffusion distance is related to the spinning conditions as,

$$\langle x^2 \rangle^{\frac{1}{2}} = \sqrt{\frac{4\,D\,L_{bath}\,A_{nozz}}{Q\ \ DR}}$$

The intial gel fibre diameter is also determined by the spinning conditions as the gel fibre area is given by the spinneret nozzle area divided by the draw ratio. Hence, the initial gel fibre diameter, d_{gf}, is given by

$$d_{gf} = \sqrt{\frac{4\,A_{nozz}}{\pi\ \ DR}}$$

Finally, an estimate of the effectiveness of washing can be given as the root mean squared diffusion distance divided by the gel fibre diameter as,

$$\frac{\langle x^2 \rangle^{\frac{1}{2}}}{d_{gf}} = \sqrt{\frac{4\,D\,L_{bath}\,A_{nozz}}{Q\ \ DR}}\sqrt{\frac{\pi\ \ DR}{4\ \ A_{nozz}}} = \sqrt{\frac{L_{bath}\,\pi\ \ D}{Q}}$$

as stated in equation (4).
